#  “Reducing unnecessary testing in a CPOE system through implementation of a targeted CDS intervention”

**DOI:** 10.1186/1472-6947-13-43

**Published:** 2013-04-08

**Authors:** Donald L Levick, Glenn Stern, Chad D Meyerhoefer, Aaron Levick, David Pucklavage

**Affiliations:** 1 Office of Chief Medical Information Officer, Lehigh Valley Health Network, Cedar Crest and I-78, PO Box 689, Allentown, PA, 18105, USA; 2 Chasm Crossing Consulting, 1470 Limeport Pike, Coopersburg, PA, USA; 3 Rauch Business Center, 621 Taylor Street, Bethlehem, PA, 18015, USA; 4 Department of Physics, Cornell University, 109 Clark Hall, Ithaca, NY, 14853, USA; 5 Information Services, Lehigh Valley Health Network, Cedar Crest and I-78, PO Box 689, Allentown, PA, 18105, USA

**Keywords:** Clinical decision support, Computerized physician order entry, Medical error, BNP testing, Alert fatigue, Sociotechnical, Unintended consequences, Appropriate testing

## Abstract

**Background:**

We describe and evaluate the development and use of a Clinical Decision Support (CDS) intervention; an alert, in response to an identified medical error of overuse of a diagnostic laboratory test in a Computerized Physician Order Entry (CPOE) system. CPOE with embedded CDS has been shown to improve quality of care and reduce medical errors. CPOE can also improve resource utilization through more appropriate use of laboratory tests and diagnostic studies. Observational studies are necessary in order to understand how these technologies can be successfully employed by healthcare providers.

**Methods:**

The error was identified by the Test Utilization Committee (TUC) in September, 2008 when they noticed critical care patients were being tested daily, and sometimes twice daily, for B-Type Natriuretic Peptide (BNP). Repeat and/or serial BNP testing is inappropriate for guiding the management of heart failure and may be clinically misleading. The CDS intervention consists of an expert rule that searches the system for a BNP lab value on the patient. If there is a value and the value is within the current hospital stay, an advisory is displayed to the ordering clinician. In order to isolate the impact of this intervention on unnecessary BNP testing we applied multiple regression analysis to the sample of 41,306 patient admissions with at least one BNP test at LVHN between January, 2008 and September, 2011.

**Results:**

Our regression results suggest the CDS intervention reduced BNP orders by 21% relative to the mean. The financial impact of the rule was also significant. Multiplying by the direct supply cost of $28.04 per test, the intervention saved approximately $92,000 per year.

**Conclusions:**

The use of alerts has great positive potential to improve care, but should be used judiciously and in the appropriate environment. While these savings may not be generalizable to other interventions, the experience at LVHN suggests that appropriately designed and carefully implemented CDS interventions can have a substantial impact on the efficiency of care provision.

## Background

Computerized Physician Order Entry (CPOE) and embedded Clinical Decision Support (CDS) have been shown to improve quality of care and reduce medical errors [1-3]. CPOE and CDS can also improve resource utilization through more appropriate use of laboratory tests and diagnostic studies. While a few Random-Controlled Trials (RCTs) have addressed CDS and CPOE efficacy [2,4,5], there are inherent limitations on the external validity of such studies. As a result, observational studies are also necessary in order to understand how these technologies can be successfully employed by healthcare providers. However, documentation of provider-initiated interventions is often limited, and as a result, more studies of implementation at the point of care are needed to understand and measure CDS and CPOE effectiveness [6,7].

We describe the development and use of a CDS intervention in a CPOE system; an alert, in response to an administratively identified medical error of overuse of a diagnostic laboratory test. We discuss the complexity of effective CPOE implementation and demonstrate the benefits achieved by this quality improvement initiative through a reduction in inappropriate testing and lower medical care costs [2,7].

The medical error motivating this intervention was identified by the Test Utilization Committee (TUC) at the Lehigh Valley Health Network (LVHN), in Allentown, PA on September, 2008. The TUC noticed critical care patients were being tested daily, and sometimes twice daily, for B-Type Natriuretic Peptide (BNP). BNP is secreted by the heart in response to changes in pressure that occur in patients with congestive heart failure (CHF). It is used as a marker to gauge the change in severity of CHF over time. Repeat and/or serial BNP testing is inappropriate for guiding the management of heart failure and may in fact be clinically misleading [8]. Given that additional BNP tests during an acute exacerbation of CHF provide no relevant clinical information, it was initially unclear why so many tests were being ordered. This overuse did not likely result in adverse clinical events [9], but it did significantly increase treatment costs.

All CDS interventions in response to identified errors involve critical success factors such as a culture of acceptance of both technology and embedded ‘cognitive forcing strategies” (rules and alerts, standardized order sets, etc.) [10]. This report describes how one healthcare system designed a targeted CDS intervention to reduce unnecessary testing (and associated costs) in a CPOE system, and evaluates its effectiveness. This intervention was part of a larger initiative to create an “advanced” [11] CDS environment capacity at LVHN. Advanced CDS development moves beyond basic CPOE functions to respond to identified medical errors, evidence-based information changes, potential unintended consequences and continuous quality improvement (CQI) over time, benefiting from a supportive socio-technical infrastructure [11].

This study emerges from a growing body of literature that seeks to rigorously evaluate applications of Health IT in daily practice. A range of studies have focused on a sociotechnical approach in order to evaluate the multiple factors that contribute to effectiveness of these new tools [12-14]. Factors that have contributed the use and ability to evaluate decision support technologies include greater emphasis by providers on knowledge-management, and an increasing quantity of patient-specific data from genetic testing and electronic health records [15]. The enhanced ability of providers to analyze clinical practice patterns has also contributed to studies seeking to define what constitutes “appropriate testing” [16].

Among decision support tools, alerts and rules have received a great deal of attention in the literature due to the significant potential to improve care. CPOE systems have been shown to reduce medical errors and overall medical costs [1-3,17]. Several other studies and recent review articles [2,3] generally support the potential of CPOE and CDS to improve care, but note that most of the evidence related to effectiveness of these tools is from academic hospitals rather than community hospitals and or primary care facilities. Alerts and rules have also received a great deal of attention in the literature due to identified barriers to their acceptance and implementation [14,18-21], and unintended consequences [14,22-25]. For example, this CDS intervention, and the development of the CPOE system in general, was designed to minimize “alert fatigue” that may occur when clinicians are overwhelmed by the number of alerts [21].

### CPOE development and implementation

Lehigh Valley Health Network (LVHN) is the largest health care provider in the Lehigh Valley region of Pennsylvania with: three not-for-profit hospitals; nine health centers in four counties; primary and specialty care practices throughout the region; pharmacy, imaging and lab services; and preferred provider services. In fiscal year 2012, LVHN had over 54,000 acute admissions and 173,000 emergency department visits. The primary service area of Lehigh, Northampton and Carbon counties has a combined population of more than 800,000, making it Pennsylvania’s third most populated region. Minorities comprise more than 54% of all Allentown residents (the region’s largest city), almost 43% of whom are Hispanic or Latino.

LVHN’s Information Technology (IT) Department was an early adopter of CPOE and CDS. The network has been named one of the *100 Most Wired* and *25 Most Wireless hospitals* by Hospitals and Health Networks magazine. IT applications at LVHN are implemented through a socio-technical infrastructure that facilitates input from providers and includes an evaluative component pre and post implementation. Examples of this infrastructure include the Test Utilization Council (TUC) that identified over-testing for BNP, and enterprise wide Clinical Decision Support Committee, safety committees, and technology assessment groups. These groups and committees maintain an ongoing sensitivity to work flow issues in the implementation of CDS, including just-in-time evidence-based decision support (clinical knowledge) [15]. The discussion below demonstrates an adaptation to overcome a socio-technical barrier to effective usage of this technology—intrusion into the physician work flow.

LVHN began implementation of CPOE in 2001; by 2006, all beds were converted and CPOE use was mandatory. Concurrent with the implementation of CPOE was the installation of a closed loop medication administration system including embedded clinical decision support, automated pharmacy robot dispensing, online medication administration documentation and bar code medication administration. At LVHN, the implementation of this closed loop medication administration system is estimated to have reduced potential harmful medication errors by 80%. ^a^

The initial approach to CDS at LVHN was very deliberate. The implementation team was acutely aware of the impact that CPOE and CDS interventions had on physician workflow as well as their potential for generating alert fatigue. Consequently, interventions that would “add clicks” (such as alerts and rules) and standardized order sets were added judiciously during the early stages of the CPOE rollout—the “basic stage” [11]. As the physicians acclimated to the system, they became more receptive to an increasing number of CDS interventions. Moving physicians along this “continuum of intrusion” [26] was considered an important success factor by all stakeholders for this CDS intervention.

#### Identification of BNP over-testing as a medical error

In September 2008, the Senior Clinical Pathologist noticed critical care patients were being tested daily, and sometimes twice daily, for B-type natriuretic Peptide (BNP). The issue was brought to the TUC and prioritized as a high volume (and high cost) test requiring further investigation and intervention. Research revealed that repeat BNP testing is usually not indicated during a hospital stay [8]. The TUC recommended modifying LVHN’s existing CPOE system to employ an expert rule to alert the ordering clinician that a BNP had been performed during the current admission and that repeat testing would add no value to the clinical decision making process.

## Methods

### CDS alert development

**Figure 1:**
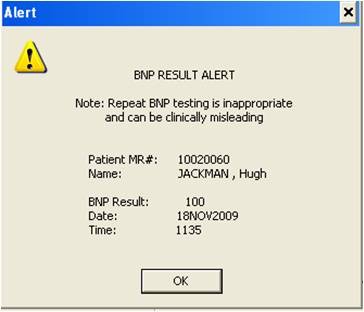
Screenshot of alert providing recent BNP result information.

**Figure 2:**
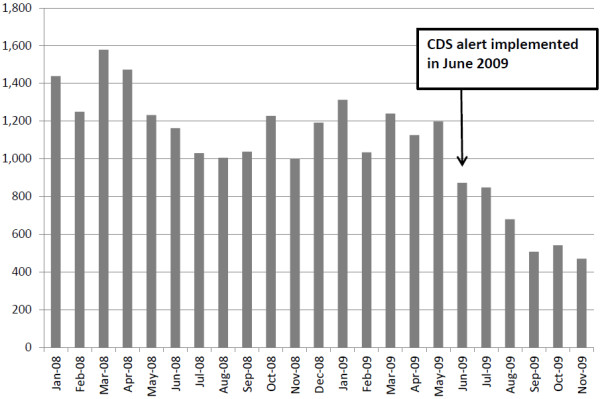
Number of monthly inpatient BNP tests, January 2008 – November, 2009.

The CDS alert was implemented through an expert rule that searches the system for a BNP lab value on the patient. If there is a value and the value is within the current hospital stay, an advisory is displayed to the ordering clinician. (See Figure 1 for a screenshot of the alert.) If there is no BNP lab value on the patient within the current episode of care, the rule evaluates pending orders for an active order for a BNP within the current episode. If an active order is found, a different advisory is displayed. If no lab value and no pending active order exist in the current episode of care, then no advisory is displayed. The alerts are advisory only and considered a “soft stop” because the clinician still has the opportunity to place the order after having been presented with relevant information. The CDS intervention for repeat BNP testing was implemented in June, 2009 and BNP ordering decreased by approximately 65% within six months of introduction of the alert. Comparing the three month period (January through March) before and after the intervention indicates that total BNP ordering on in-patient encounters decreased from 3,291 to 2,563, and the number of patients receiving two or more BNP tests during a single hospital stay decreased from 1,358 to 487 (p < .01). However, Figure 2 also reveals that there was a downward trend in BNP testing during the surrounding time period of the intervention. This trend could be the result of several factors, including behavioral change on the part of physicians or process changes caused by other aspects of the new medication administration system, as well as changes in the health and demographic characteristics of patients eligible for the BNP test.

### Multivariate statistical analysis

In order to isolate the impact of the CPOE intervention on unnecessary BNP testing, we apply multiple regression analysis to the sample of 41,306 patient admissions with at least one BNP test at LVHN between January, 2008 and September, 2011. Although the average number of tests ordered was 1.8, the distribution of BNP orders is skewed to the right with a maximum value of 31 (see Table 1). Therefore, we model the number of BNP orders using a zero-truncated negative binomial (ZTNB) distribution to account for both the truncation of the dependent variable at 1, and the skewness of the distribution [27].

**Table 1:** Descriptive statistics for analysis variables (N = 41,306)

	**Mean**	**Min**	**Max**
BNP Orders	1.787	1	31
	(SD = 1.364)		
Age ≤ 40	0.022	0	1
40 < Age ≤ 60	0.146	0	1
60 < Age ≤ 80	0.432	0	1
Age > 80	0.399	0	1
Female	0.500	0	1
White	0.905	0	1
Black	0.023	0	1
Hispanic	0.024	0	1
Other race	0.048	0	1
Emergency admission	0.849	0	1
Elective admission	0.046	0	1
Urgent admission	0.103	0	1
DCG/HCC risk score	4.347	−0.875	17.779
	(SD = 2.271)		
Private insurance	0.132	0	1
Medicare	0.635	0	1
Medicaid	0.023	0	1
Self-pay	0.007	0	1
Private managed care	0.027	0	1
Medicare managed care	0.162	0	1
Medicaid managed care	0.014	0	1
Monthly time trend	21.659	1	45
	(SD = 0.496)		
Post intervention period	0.564	0	1

We include a variety of controls in our models to account for changes over time in the characteristics of patients that may influence the propensity of physicians to order additional tests. These include indicator variables for patient age (age ≤ 40; 40 < age ≤ 60; 60 < age ≤ 80; age > 80), gender, race (white; black; Hispanic; other race), admission type (emergency; elective; urgent), and insurance type (private traditional plan; Medicare; Medicaid; self-pay; private managed care plan; Medicare managed care; Medicaid managed care). In addition, we include indicators for quartile of the inpatient cost risk distribution, where risk is based on patients’ Diagnostic Cost Group/Hierarchical Condition Category (DCG/HCC) risk scores. ^b^

Our intervention indicator is equal to 1 for all patient admissions during or after June, 2009, the month that the CPOE alert was put and place, and is equal to 0 before that date. The mean, minimum, and maximum of the intervention indicator and all of the control variables are listed in Table 1.

## Results

We estimate three ZTNB regression models in order to test the sensitivity of our results to different specifications for capturing overall trends in BNP orders not due to the intervention during the sample period. These results are reported in Table 2, which contains the marginal effects of the control variables and the intervention indicator. The marginal effects are interpreted as the impact on the number of BNP orders corresponding to a one unit change in the given variable. The standard errors of the marginal effects in parenthesis are robust to heteroscedasticity.

**Table 2:** Change in BNP orders associated with one unit change in selected variables from ZTNB regression

	**(1)**	**(2)**	**(3)**
40 < Age ≤ 60	0.125**	0.115**	0.114**
	(0.055)	(0.055)	(0.056)
60 < Age ≤ 80	0.237***	0.235***	0.247***
	(0.052)	(0.052)	(0.053)
Age > 80	0.315***	0.310***	0.326***
	(0.055)	(0.055)	(0.057)
Female	−0.005	−0.006	−0.006
	(0.011)	(0.011)	(0.012)
Black	−0.010	−0.012	−0.008
	(0.040)	(0.040)	(0.041)
Hispanic	−0.104***	−0.085**	−0.010***
	(0.035)	(0.037)	(0.036)
Other race	0.192***	0.190***	0.196***
	(0.034)	(0.034)	(0.034)
Elective admission	−0.094***	−0.085***	−0.089***
	(0.028)	(0.028)	(0.028)
Urgent admission	−0.005	0.003	0.007
	(0.019)	(0.019)	(0.020)
1^st^ risk score quartile	−0.481***	−0.499***	−0.506***
	(0.013)	(0.014)	(0.013)
2^nd^ risk score quartile	−0.322***	−0.337***	−0.338***
	(0.013)	(0.013)***	(0.013)
3^rd^ risk score quartile	−0.157***	−0.170***	−0.167***
	(0.013)	(0.013)	(0.014)
Private insurance	−0.031	−0.031	−0.024
	(0.021)	(0.021)	(0.021)
Medicaid	0.088*	0.074	0.088*
	(0.050)	(0.048)	(0.050)
Self-pay	0.051	0.051	0.034
	(0.084)	(0.086)	(0.085)
Private managed care	0.002	0.002	0.013
	(0.041)	(0.041)	(0.043)
Medicare managed care	−0.030**	−0.028*	−0.030**
	(0.015)	(0.015)	(0.015)
Medicaid managed care	−0.001	−0.008	−0.002
	(0.069)	(0.061)	(0.062)
Post intervention period	−0.598***	−0.460***	−0.378***
	(0.028)	(0.030)	(0.056)
*Time trend variables*			
Monthly trend	X	X	
Monthly trend squared		X	
Month indicators			X

Notes: Standard errors in parenthesis; *** p<0.01, **p<0.05, *p<0.1. Base categories include: age ≤ 40; white; emergency admission; 4^th^ (highest) risk score quartile; and Medicare.

In the first specification in column 1 we include a single monthly time trend variable to capture aggregate trends in BNP orders net of the CDS intervention. The estimates from this specification suggest that the intervention reduced the number of BNP orders by 0.6 per inpatient admission, on average. However, when we include additional variables to more flexibility control for aggregate trends over time, this estimate is reduced by over 20%. In addition to a linear monthly time trend, the specification in column 2 also contains the square of the time trend, while the specification in column 3 includes separate indicator variables for each month of the sample. Among these models, we prefer the third and most flexible specification, which indicates that the intervention reduced the number of BNP tests by 0.4 per admission. This effect is precisely estimated, and represents a reduction in BNP orders of 21% relative to the mean.

The financial impact of the rule was also significant. In the three quarters prior to the implementation of the intervention there were 11,209 BNP tests ordered at LVHN. Based on our statistical model, we project that the intervention reduced BNP orders by 21%, or 2,454 tests over three quarters. Multiplying by the direct supply cost of $28.04 per test, this suggests the intervention saved approximately $92,000 per year. These savings are based on supply cost only, and do not account for the increased capacity for the laboratory to perform other tests. As a result, the full annual long run benefits of the intervention are greater than our estimate of supply cost savings, after the short term costs of implementing the intervention are recovered.

## Discussion

While there are few observational studies that evaluate the effectiveness of similar CDS interventions, Bates et al [3]. found using a randomized controlled trial that computerized reminders about redundant laboratory tests significantly reduced the final number of ordered tests. There is also a paucity of research on the impact of CDS and CPOE on costs. A comprehensive 2010 research review of the impact of CDS on test ordering and appropriate utilization in the UK was not able to identify any specific studies on reduced costs from interventions that reduced overuse of testing [4]. Nonetheless, the importance of studies that determine the impact of CDS and CPOE on cost is well recognized [2,4,7,11,28] A recent meta analysis of the relatively small number of studies currently available that do address the cost consequences of CPOE and CDS, found a significant amount of variability in the level of cost savings; ranging from $6,000 to $84,194 [29].

Much of the previous literature on CPOE addresses the unintended consequences that result from the implementation of new technologies, the most serious of which is e-iatrogenesis. Because there is no evidence that this over-testing error caused harm to patients, it is not an example of e-iatrogenesis. Furthermore, it is not clear whether over-testing for BNP was in fact an unintended consequence of CPOE. [22-25] In that case it may or may not be classified as a “new kind of error” [24] or an error made because of the existence of CPOE. LVHN front line staff concluded that the over-testing occurred because either the ordering physician did not understand the time-period of the reliability of the test, or the physician was unable to find previous orders/ results in the electronic record (or did not take the time to search the system). If the former hypothesis is accurate then the experience of LVHN demonstrates the benefit of CPOE in uncovering previously unrecognized instances of inefficient care, and the ability of complementary CDS technologies to increase the efficiency of care provision and lower costs. In contrast, if the latter hypothesis is correct, LVHN’s experience demonstrates how CDS can be used to correct an unintended consequence of CPOE .In both cases the CDS intervention led to a positive outcome, but the two underlying reasons for the over-use error have very different implications for the efficacy of CPOE. This highlights the need for future research studies that use a mix of qualitative and quantitative methods to fully characterize and evaluate the use of new technologies [11-13,15]. Irrespective of the source of the over-use error, LVHN’s method of identifying and correcting the error can be replicated by other health care networks with similar IT systems and socio-technical infrastructures.

## Conclusion

In the specific case of CDS, the use of alerts has great potential to improve care, but should be used judiciously and in the appropriate environment [11,14,20,26,30]. The intervention implemented at LVHN to reduce unnecessary testing was effective because: 1) It alerted physicians that further BNP testing is potentially misleading; 2) It addressed an information failure associated with the EMR (previous episode of care results were not visible once results were posted) and; 3) The intervention was implemented and evaluated in the context of an advanced [11] CPOE system facilitating this cycle of implementation and evaluation. Although we cannot determine how each of these factors independently contributed to the success of the intervention, our statistical analysis indicates that they collectively resulted in the reduction of BNP testing by 21%; saving LVHN over $92,000 per year. While these savings may not be generalizable to other interventions, the experience at LVHN suggests that appropriately designed and carefully implemented CDS interventions can have a substantial impact on the efficiency of care provision.

## Endnotes

^a^Lehigh Valley Health Network, Internal Report, 2011

^b^DCG/HCC risk scores are derived from data on patient age, sex, and physician-reported diagnosis codes (ICD-9-CM). They have been validated as a proper measure of risk adjustment in the inpatient setting (Ash et al., 2003; Petersen, Pietz, Woodard & Byrne, 2005) and are also used by CMS to risk adjust Medicare payments to private insurers under Part C (Pope et al., 2004; Pope et al., 2000).

## Competing interest

The author(s) declare that they have no competing interests.

## Authors’ contributions

DL was involved in the development of the study, drafting and editing of the manuscript. DP participated in the development of the study and the initial draft of the document. AL performed the literature search and in the development of the manuscript. GS provided the theoretical frameworks and performed much of the editing of the manuscript. CM provided statistical analysis and contributed to the restructuring of the manuscript. All authors read and approved the final manuscript.

## Pre-publication history

The pre-publication history for this paper can be accessed here:

http://www.biomedcentral.com/1472-6947/13/43/prepub
